# Crystal structures of isotypic poly[bis­(benz­imid­azolium) [tetra-μ-iodido-stannate(II)]] and poly[bis­(5,6-di­fluoro­benzimidazolium) [tetra-μ-iodido-stannate(II)]]

**DOI:** 10.1107/S1600536814019151

**Published:** 2014-09-10

**Authors:** Iwan Zimmermann, Tony D. Keene, Jürg Hauser, Silvio Decurtins, Shi-Xia Liu

**Affiliations:** a Department of Chemistry and Biochemistry, University of Bern, Freiestrasse 3, 3012 Bern, Switzerland

**Keywords:** crystal structure, benzimidazolium, tin(II) iodide layers, perovskite layer

## Abstract

The isostructural title compounds, {(C_7_H_7_N_2_)_2_[SnI_4_]}_*n*_, (1), and {(C_7_H_5_F_2_N_2_)_2_[SnI_4_]}_*n*_, (2), show a layered perovskite-type structure composed of anionic {[SnI_4_]^2−^}_*n*_ sheets parallel to (100), which are decorated on both sides with templating benzimidazolium or 5,6-di­fluoro­benzimidazolium cations, respectively. These planar organic heterocycles mainly form N—H⋯I hydrogen bonds to the terminal I atoms of the corner-sharing [SnI_6_] octa­hedra (point group symmetry 2) from the inorganic layer, but not to the bridging ones. This is in contrast to most of the reported structures of related compounds where ammonium cations are involved. Here hydrogen bonding to both types of iodine atoms and thereby a distortion of the inorganic layers to various extents is observed. For (1) and (2), all Sn—I—Sn angles are linear and no out-of-plane distortions of the inorganic layers occur, a fact of relevance in view of the material properties. The arrangement of the aromatic cations is mainly determined through the direction of the N—H⋯I hydrogen bonds. The coherence between organic bilayers along [100] is mainly achieved through van der Waals inter­actions.

## Abstract 2

The bicyclic aromatic benzimidazolium cation stabilizes the layered perovskite structure comprising inorganic {[SnI_4_]^2−^}_*n*_ sheets. A di­fluoro-substitution of the organic cation demonstrates the structural versatility of the new approach.

## Chemical context   

The title compounds, (1) and (2), belong to an extensive family of materials exhibiting a perovskite-type structure, which can vary with respect to the dimensionality of its extended inorganic framework, ranging from two-dimensional, [*MX*_4_]_*n*_^2*n*−^, to three-dimensional, [*MX*_3_]*_n_^n^* ^−^ (Mitzi, 1999 ▶, 2001 ▶, 2004 ▶; Mitzi *et al.*, 2001 ▶; Zhengtao *et al.*, 2003**a* ▶,b* ▶). The former case is exemplified by anionic [*MX*_4_]_*n*_^2*n*−^ sheets (*M* = divalent metal ion; *X* = halogen) of corner-sharing *MX*_6_ octa­hedra, which are separated by bilayers of organic cations.
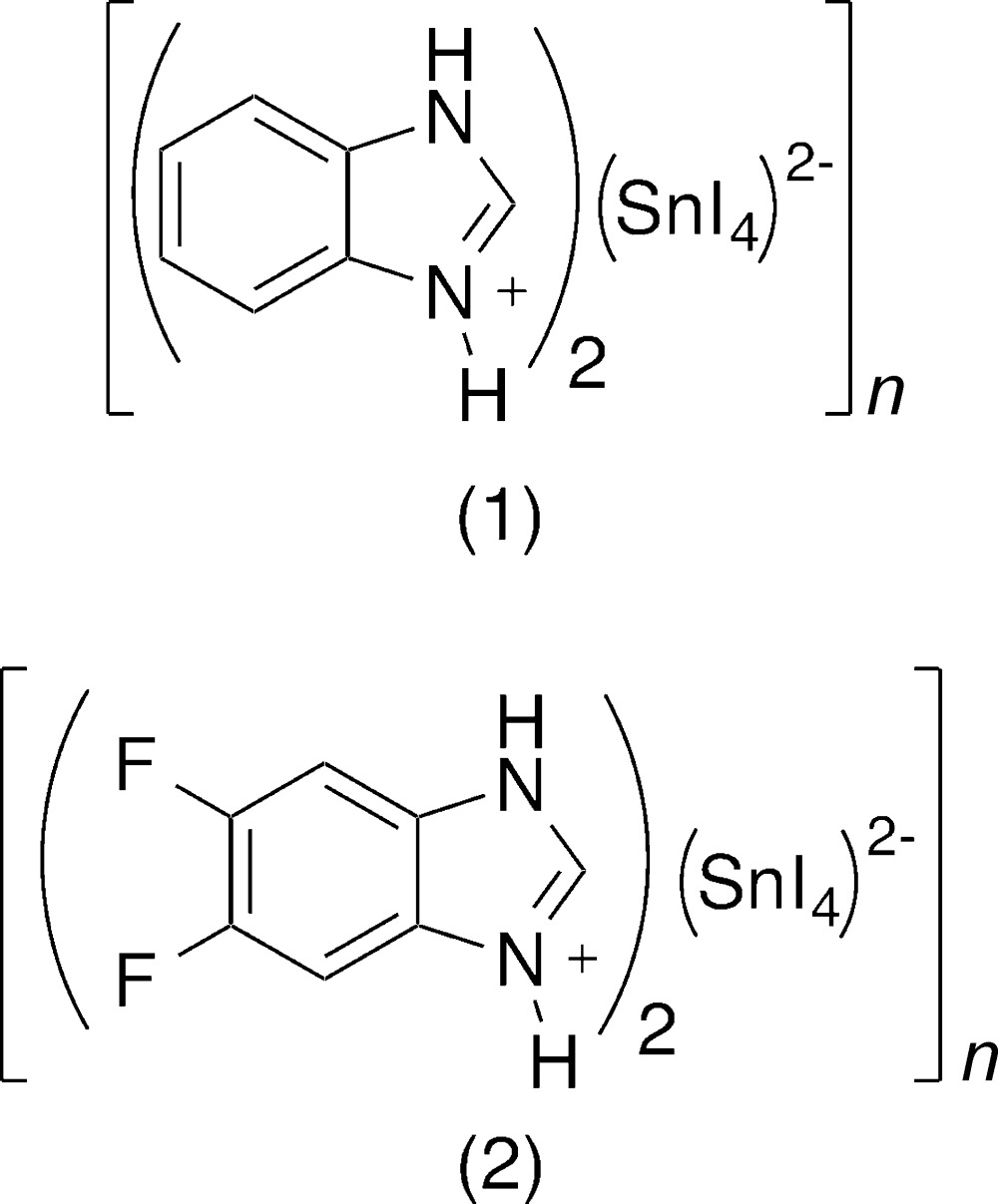


 For most reported layered perovskites, these organic mol­ecules are terminated with one or two protonated primary amine groups. Thereby, the ammonium head(s) form N—H⋯*X* hydrogen bonds to any of the bridging and terminal halogen atoms in the inorganic layers (Mitzi *et al.*, 2002 ▶; Mercier *et al.*, 2004 ▶; Sourisseau *et al.*, 2007 ▶; Pradeesh *et al.*, 2013 ▶). In the actual case, however, as a novel aspect, the bicyclic aromatic benzimidazole unit is introduced as an organic part. There are numerous general examples of benzimidazole acting as a neutral ligand (Keene *et al.*, 2010 ▶) and similarly in its protonated form (Mouchaham *et al.*, 2010 ▶). In this context, the present study explicitly demonstrates that benzimidazolium cations and corresponding derivatives can stabilize the layered perovskite structure as well, while fitting perfectly into the ‘footprint’ provided by the inorganic framework. This observation bears importance since the extent of the in- and out-of-plane angular distortions, twisting and buckling of the anionic sheets, is largely determined by the relative charge density, steric requirements and hydrogen-bonding pattern of the organic cations (Knutson & Martin, 2005 ▶; Takahashi *et al.*, 2007 ▶). These distortions correlate with the band gaps of the perovskite-type semiconductors. It is inter­esting to note that perovskite-based solar cells have recently been catapulted to the cutting edge of thin-film photovoltaic research (Hao *et al.*, 2014 ▶; Marchioro *et al.*, 2014 ▶). Consequently, the chemical variability which comes with the imidazolium cation, especially the range of possible substitutions on its mol­ecular skeleton, gives an additional structural diversity to this class of compounds. As a case in point, consider the di­fluoro-substituted compound (2) which renders not only modified van der Waals inter­actions for the organic bilayers, but also tailors the ‘chemistry’ of the crystal surfaces.

## Structural commentary   

**Figure 1:**
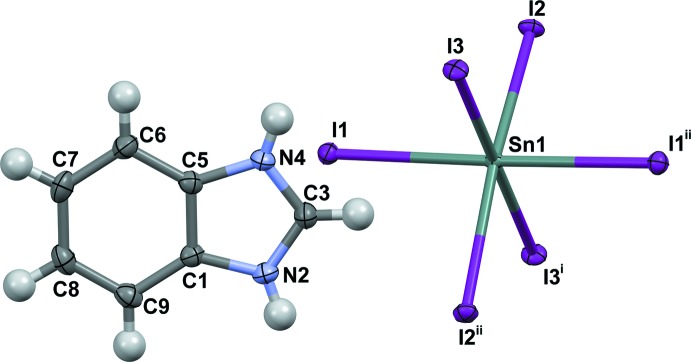
The main building units of (1), showing atom labeling and displacement ellipsoids drawn at the 50% probability level. [Symmetry codes: (i) *x*, *y* + 1, *z*; (ii) −*x*, *y*, −*z* + 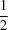.]

**Figure 3:**
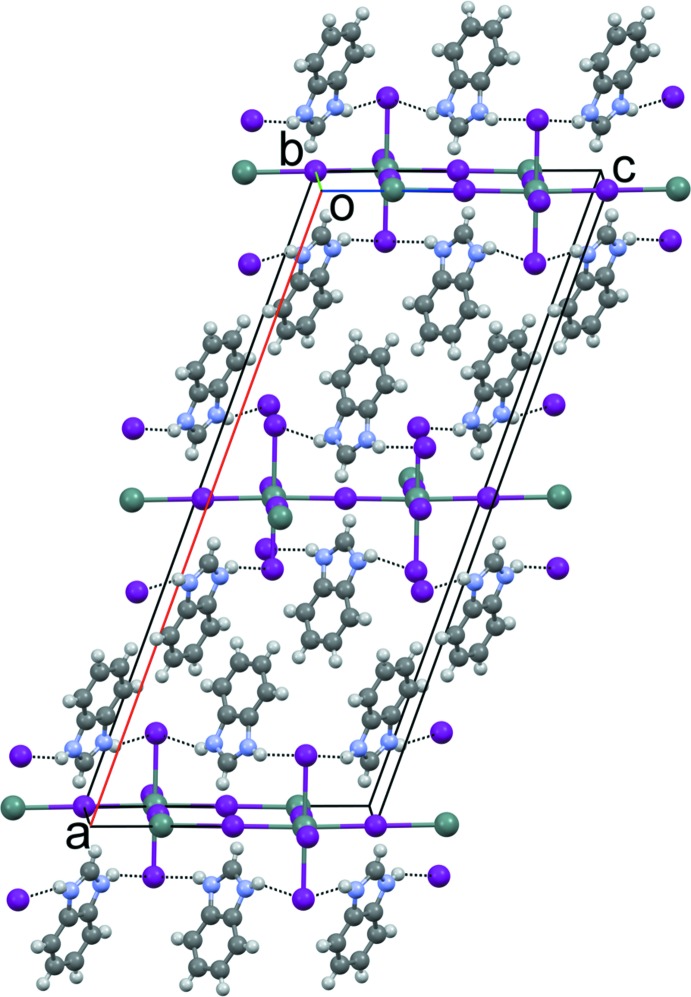
The crystal packing of compound (1) viewed along [010]. N—H⋯I hydrogen bonds are shown as dashed lines.

**Figure 5:**
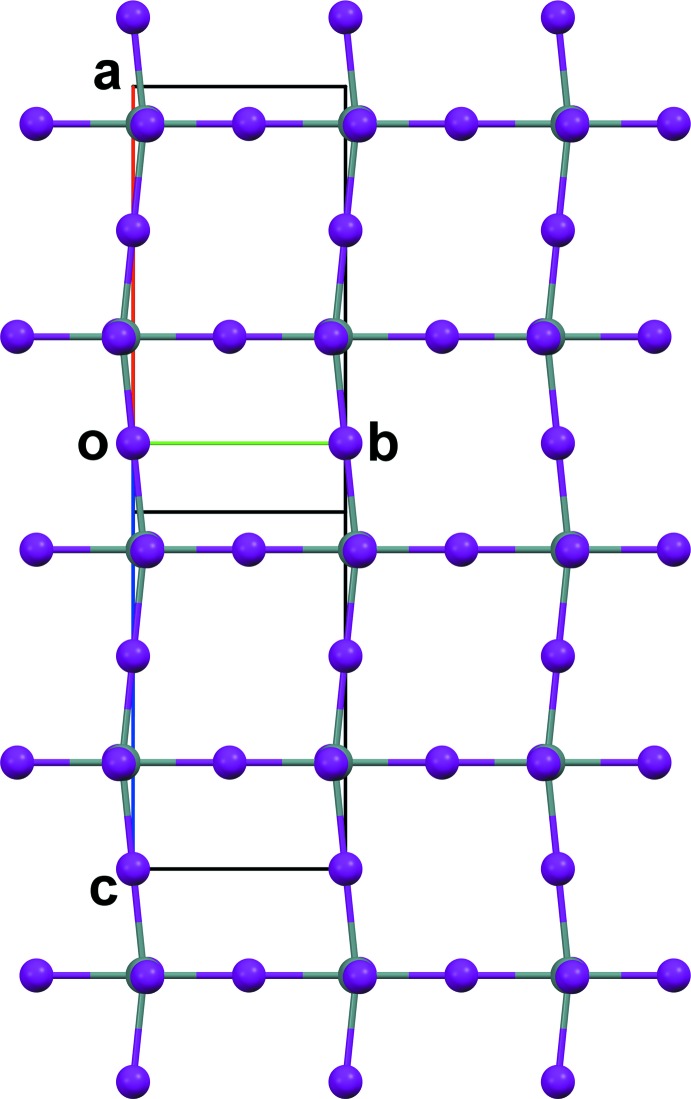
View along the a* axis of a tin iodide layer of (2). For clarity, the atoms are represented as spheres with uniform sizes selected for each atom type.

**Figure 6:**
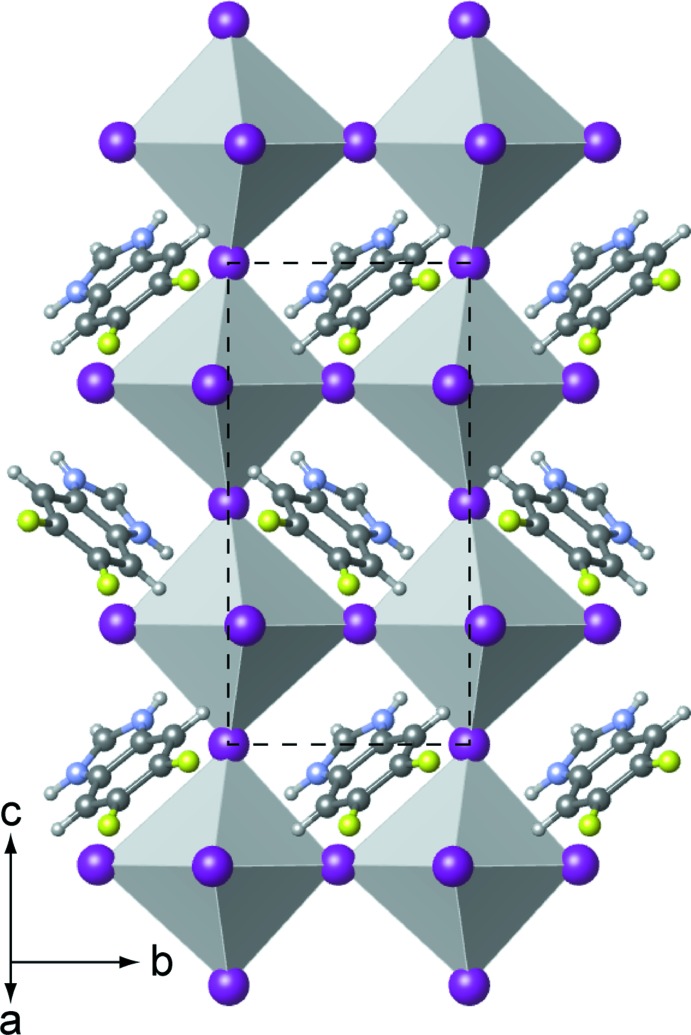
View along the a* axis of a double layer of tin iodide and the organic cations of (2). For clarity, the [SnI_6_] octa­hedra are shown as polyhedra, the atoms of the organic cations are represented as spheres with uniform sizes selected for each atom type.

Compounds (1) and (2) are isostructural. Their asymmetric units, Figs. 1 ▶ and 2 ▶, consist of an Sn^2+^ cation situated on a twofold rotation axis (Wyckoff position 4*e*), three iodine atoms [one in a general position, one on an inversion centre (4*a*) and one on a twofold rotation axis (4*e*)] and a benz­imid­azolium or 5,6-di­fluoro­benzimidazolium cation, respectively. The main building blocks of the structure are corner-sharing [SnI_6_] octa­hedra, which form planar sheets with formula {[SnI_4_]^2−^}_*n*_ which extend parallel to (100). The negative charge of these layers is compensated by the organic cations, which are on both sides of the layer, attached by strong hydrogen-bonding and Coulombic inter­actions (Figs. 3 ▶ and 4 ▶). This structural motif can be regarded as an *A–B–A* layer system, where *A* represents the aromatic cation and *B* the tin iodide layer. The coherence between organic bilayers along [100] is mainly achieved through van der Waals inter­actions. The Sn—I bond lengths for (1) range from 3.0626 (3) Å to 3.1607 (3) Å [(2): 3.0491 (5) Å to 3.1596 (3) Å], with no distinct pattern for bridging compared to terminal iodine atoms (Tables 1 ▶ and 2 ▶). These values are in agreement with those reported previously for related tin iodide perovskite structures, as for example [(C_4_H_9_NH_3_)_2_[SnI_4_]], where the bond lengths range from 3.133 Å to 3.16 Å (Mitzi, 1996 ▶). The I—Sn—I angles of the [SnI_6_] octa­hedra in the title structures deviate slightly from the ideal octa­hedral geometry. With 83.886 (4)° for (1) [(2): 84.077 (6)°], the I2—Sn1—I3 angle has the largest difference. On the other hand, all Sn—I—Sn angles are linear, which leads to the formation of an almost rectangular grid (Fig. 5 ▶). There is no out-of-plane distortion of the inorganic sheet. The arrangement of the aromatic cations is mainly determined through the direction of N—H⋯I hydrogen bonds to the apical iodine atoms (Tables 3 ▶ and 4 ▶; Figs. 3 ▶ and 4 ▶). There is no N—H⋯I_bridging_ contact smaller than the sum of the respective van der Waals radii (H: 1.2, I: 1.98 Å; Bondi, 1964 ▶). This is in contrast to primary ammonium cations, which form hydrogen bonds to both apical and bridging iodine atoms. The shortest H⋯I_bridging_ distance is C3—H3⋯I2 with 3.12 Å for (1) [(2): 3.19 Å] close to the sum of van der Waals radii. Adjacent cations within an organic layer show a plane-to-plane distance of 3.786 Å for (1) [(2): 3.730 Å] (Fig. 6 ▶). The shortest contact distances between the organic bilayers for both compounds are close to the sums of the van der Waals radii [C8⋯H6^i^ 2.801 Å in (1) and F8⋯H9^ii^ 2.557 Å in (2); (i): 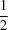 − *x*, −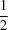 + *y*, 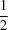 − *z*; (ii): 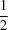 − *x*, 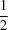 − *y*, −*z*]. The larger size of the fluorine atom in comparison to the hydrogen atom is reflected in a larger *A–B–A* layer spacing of 14.407 Å for (2) compared to 13.950 Å for (1).

## Database survey   

In the Cambridge Structural Database (Version 5.35, last update November 2013; Allen, 2002 ▶) no structures of compounds containing a (benz)imidazolium cation for layered perovskites are listed, making the two examples presented herein the only ones reported so far.

## Synthesis and crystallization   

Compound (1) was synthesized and crystallized by a solvothermal method using a mixture of tin(II) iodide and benz­imidazole in a 1:2 molar ratio. In a 50 ml round-bottom flask, 4 ml concentrated HI (57 wt. %, stabilized with hypo­phospho­rous acid) was mixed with 2 mmol (0.236 g) benz­imidazole. After stirring for one minute, this solution was added to a sample flask containing 1 mmol (0.372 g) tin(II) iodide. The reaction flask was put in a 23 ml Teflon container. The reaction was conducted at 363 K for ten h after which the autoclave was slowly cooled (1 K/h) to room temperature. Thin, black plate-like crystals were obtained. The synthetic procedure for (2) was identical to that for (1), only using 0.5 mmol (0.77 g) 5,6-di­fluoro­benzimidazole and 0.25 mmol (0.093 g) tin(II) iodide as starting materials. Thin, black plate-like crystals were obtained.

## Refinement   

Crystal data, data collection and structure refinement details are summarized in Table 5 ▶. The N-H hydrogen atoms were located in difference Fourier maps and were freely refined. The C-bound hydrogen atoms were included in calculated positions and treated as riding atoms with C—H = 0.95 Å. The isotropic displacement parameters of all H atoms were constrained to 1.2*U*_eq_ of their parent atoms. The crystal of compound (2) was a non-merohedral twin. The two twin components were related by a twofold rotation about the c* axis. The data from both twin components were integrated to give 8236 and 7625 non-overlapped reflections for twin components 1 and 2, respectively, plus 13836 overlapping reflections from both twin components. Symmetry-equivalent reflections were merged. The major twin fraction, component 1, refined to 0.6870 (12).

## Supplementary Material

Crystal structure: contains datablock(s) 1, 2. DOI: 10.1107/S1600536814019151/wm5043sup1.cif

Structure factors: contains datablock(s) 1. DOI: 10.1107/S1600536814019151/wm50431sup2.hkl

Structure factors: contains datablock(s) 2. DOI: 10.1107/S1600536814019151/wm50432sup3.hkl

CCDC references: 1021082, 1021083

Additional supporting information:  crystallographic information; 3D view; checkCIF report

## Figures and Tables

**Figure 2:**
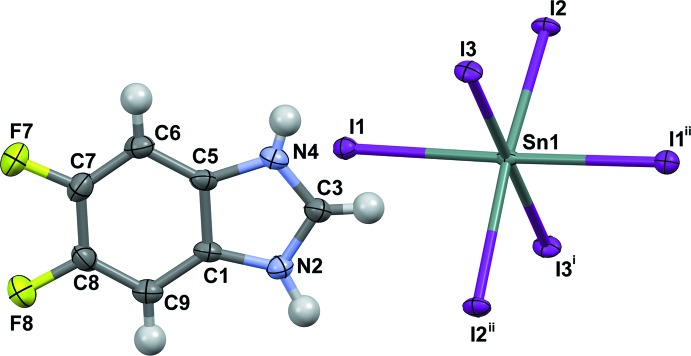
The main building units of (2), showing atom labeling and displacement ellipsoids drawn at the 50% probability level. [Symmetry codes: (i) *x*, *y* + 1, *z*; (ii) −*x*, *y*, −*z* + 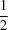.]

**Figure 4:**
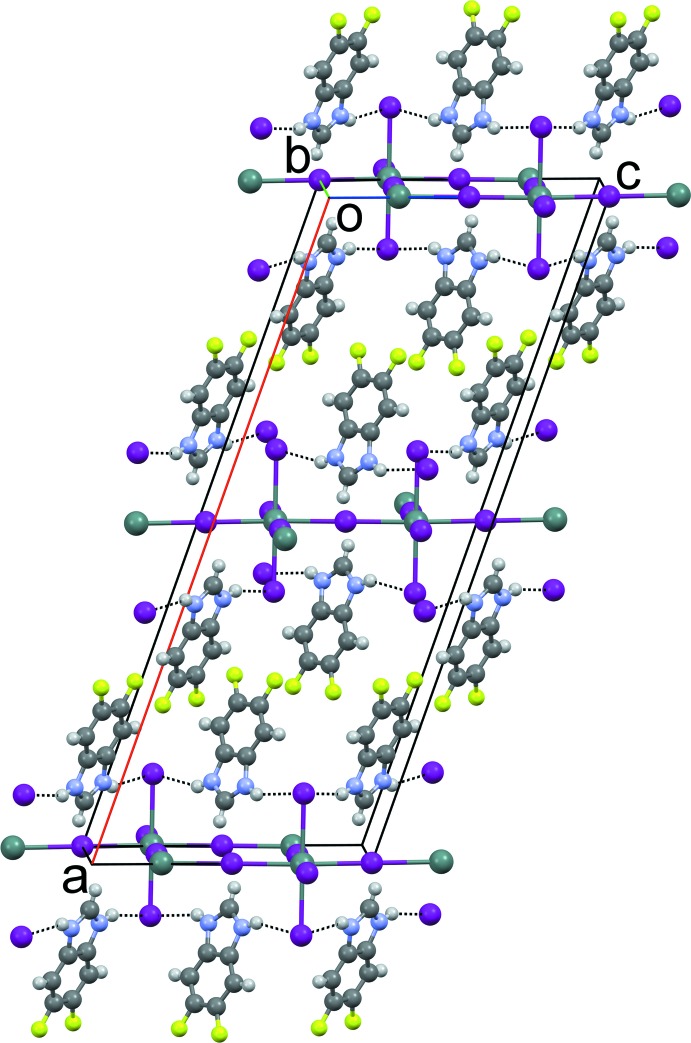
The crystal packing of compound (2) viewed along [010]. N—H⋯I hydrogen bonds are shown as dashed lines.

**Table 1:** Selected geometric parameters (Å, °) for (1)

Sn1—I1	3.1571 (2)	Sn1—I3	3.1607 (3)
Sn1—I2	3.1242 (1)	Sn1—I3^i^	3.0626 (3)
			
I1—Sn1—I2	89.357 (3)	I2—Sn1—I3	83.886 (4)
I1—Sn1—I2^ii^	90.984 (3)	I1—Sn1—I3^i^	88.396 (4)
I1—Sn1—I1^ii^	176.793 (9)	I2—Sn1—I3^i^	96.114 (4)
I2—Sn1—I2^ii^	167.773 (7)	I3—Sn1—I3^i^	180.0
I1—Sn1—I3	91.604 (4)		

Symmetry codes: (i) 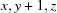; (ii) 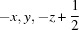.

**Table 2:** Selected geometric parameters (Å, °) for (2)

Sn1—I1	3.1596 (3)	Sn1—I3	3.1310 (5)
Sn1—I2	3.1129 (1)	Sn1—I3^i^	3.0491 (5)
			
I1—Sn1—I2	89.374 (6)	I2—Sn1—I3	84.077 (6)
I1—Sn1—I2^ii^	90.984 (6)	I1—Sn1—I3^i^	88.269 (7)
I1—Sn1—I1^ii^	176.539 (14)	I2—Sn1—I3^i^	95.923 (6)
I2—Sn1—I2^ii^	168.154 (12)	I3—Sn1—I3^i^	180.0
I1—Sn1—I3	91.731 (7)		

Symmetry codes: (i) 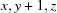; (ii) 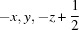.

**Table 3:** Hydrogen-bond geometry (Å, °) for (1)

*D*—H⋯*A*	*D*—H	H⋯*A*	*D*⋯*A*	*D*—H⋯*A*
N2—H2⋯I1^iii^	0.81 (3)	2.85 (3)	3.615 (2)	158 (3)
N4—H4⋯I1^i^	0.85 (3)	2.86 (3)	3.630 (2)	151 (2)

Symmetry codes: (i) 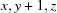; (iii) 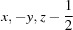.

**Table 4:** Hydrogen-bond geometry (Å, °) for (2)

*D*—H⋯*A*	*D*—H	H⋯*A*	*D*⋯*A*	*D*—H⋯*A*
N2—H2⋯I1^iii^	0.95 (6)	2.79 (6)	3.610 (4)	145 (4)
N4—H4⋯I1^i^	0.75 (5)	2.88 (6)	3.587 (4)	157 (6)

Symmetry codes: (i) 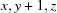; (iii) 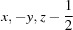.

**Table 5:** Experimental details

	(1)	(2)
Crystal data		
Chemical formula	(C_7_H_7_N_2_)_2_[SnI_4_]	(C_7_H_5_F_2_N_2_)_2_[SnI_4_]
*M*_r_	864.58	936.55
Crystal system, space group	Monoclinic, *C*2/*c*	Monoclinic, *C*2/*c*
Temperature (K)	123	123
*a*, *b*, *c* (Å)	29.6316 (5), 6.22328 (10), 12.4258 (2)	31.3825 (6), 6.18011 (12), 12.38507 (13)
β (°)	109.6798 (8)	109.3241 (7)
*V* (Å^3^)	2157.55 (6)	2266.72 (7)
*Z*	4	4
Radiation type	Mo *K*α	Mo *K*α
μ (mm^−1^)	6.91	6.61
Crystal size (mm)	0.15 × 0.10 × 0.05	0.33 × 0.33 × 0.01

Data collection		
Diffractometer	Bruker APEXII CCD	Bruker APEXII CCD
Absorption correction	Multi-scan (*SADABS*; Bruker, 2001 ▶)	Multi-scan (*TWINABS*; Bruker, 2001 ▶)
*T*_min_, *T*_max_	0.570, 0.747	0.322, 0.522
No. of measured, independent and observed [*I* > 2σ(*I*)] reflections	24695, 3713, 3222	29697, 5792, 5179
*R*_int_	0.033	?
(sin θ/λ)_max_ (Å^−1^)	0.772	0.768

Refinement		
*R*[*F* ^2^ > 2σ(*F* ^2^)], *wR*(*F* ^2^), *S*	0.022, 0.045, 1.06	0.035, 0.124, 1.07
No. of reflections	3713	5792
No. of parameters	113	132
H-atom treatment	H atoms treated by a mixture of independent and constrained refinement	H atoms treated by a mixture of independent and constrained refinement
Δρ_max_, Δρ_min_ (e Å^−3^)	0.70, −1.15	1.95, −1.74

Computer programs: *APEX2* and *SAINT-Plus* (Bruker, 2001 ▶), *SIR97* (Altomare *et al.*, 1999 ▶), *SHELXL2014* (Sheldrick, 2008 ▶), *Mercury* (Macrae *et al.*, 2008 ▶), *VESTA* (Momma & Izumi, 2011 ▶) and *publCIF* (Westrip, 2010 ▶).

## supplementary crystallographic information

### Crystal data

**Table:**

(C_7_H_5_F_2_N_2_)_2_[SnI_4_]	*F*(000) = 1680
*M**_r_* = 936.55	*D*_x_ = 2.744 Mg m^−^^3^
Monoclinic, *C*2/*c*	Mo *K*α radiation, λ = 0.71073 Å
*a* = 31.3825 (6) Å	Cell parameters from 9949 reflections
*b* = 6.18011 (12) Å	θ = 5.5–65.4°
*c* = 12.38507 (13) Å	µ = 6.61 mm^−^^1^
β = 109.3241 (7)°	*T* = 123 K
*V* = 2266.72 (7) Å^3^	Plate, black
*Z* = 4	0.33 × 0.33 × 0.01 mm

### Data collection

**Table:**

Bruker APEXII CCD diffractometer	5792 independent reflections
Radiation source: fine-focus sealed tube	5179 reflections with *I* > 2σ(*I*)
Graphite monochromator	θ_max_ = 33.1°, θ_min_ = 2.8°
rotation method scans	*h* = −47→43
Absorption correction: multi-scan (*TWINABS*; Bruker, 2001)	*k* = 0→9
*T*_min_ = 0.322, *T*_max_ = 0.522	*l* = 0→18
29697 measured reflections	

### Refinement

**Table:**

Refinement on *F*^2^	0 restraints
Least-squares matrix: full	Hydrogen site location: difference Fourier map
*R*[*F*^2^ > 2σ(*F*^2^)] = 0.035	H atoms treated by a mixture of independent and constrained refinement
*wR*(*F*^2^) = 0.124	*w* = 1/[σ^2^(*F*_o_^2^) + (0.0948*P*)^2^] where *P* = (*F*_o_^2^ + 2*F*_c_^2^)/3
*S* = 1.07	(Δ/σ)_max_ = 0.001
5792 reflections	Δρ_max_ = 1.95 e Å^−^^3^
132 parameters	Δρ_min_ = −1.74 e Å^−^^3^

### Special details

**Table:**

Geometry. All e.s.d.'s (except the e.s.d. in the dihedral angle between two l.s. planes) are estimated using the full covariance matrix. The cell e.s.d.'s are taken into account individually in the estimation of e.s.d.'s in distances, angles and torsion angles; correlations between e.s.d.'s in cell parameters are only used when they are defined by crystal symmetry. An approximate (isotropic) treatment of cell e.s.d.'s is used for estimating e.s.d.'s involving l.s. planes.
Refinement. Refined as a 2-component twin.

### Fractional atomic coordinates and isotropic or equivalent isotropic displacement parameters (Å^2^)

**Table:**

	*x*	*y*	*z*	*U*_iso_*/*U*_eq_	
Sn1	0.0000	0.05198 (5)	0.2500	0.01458 (10)	
I1	0.10663 (2)	0.06742 (4)	0.33581 (3)	0.02019 (10)	
I2	0.0000	0.0000	0.0000	0.02079 (11)	
I3	0.0000	−0.45465 (5)	0.2500	0.02092 (11)	
C1	0.14859 (19)	0.4618 (7)	0.0571 (4)	0.0227 (9)	
N2	0.10624 (15)	0.3704 (7)	0.0285 (3)	0.0249 (8)	
H2	0.092 (2)	0.260 (10)	−0.025 (5)	0.030*	
C3	0.08158 (18)	0.4824 (8)	0.0780 (5)	0.0271 (10)	
H3	0.0511	0.4521	0.0709	0.033*	
N4	0.10586 (15)	0.6417 (7)	0.1383 (3)	0.0251 (8)	
H4	0.099 (2)	0.714 (9)	0.178 (5)	0.030*	
C5	0.14871 (16)	0.6384 (8)	0.1291 (4)	0.0232 (9)	
C6	0.18595 (18)	0.7714 (8)	0.1732 (4)	0.0283 (10)	
H6	0.1860	0.8918	0.2210	0.034*	
C7	0.22244 (18)	0.7166 (9)	0.1429 (5)	0.0319 (11)	
F7	0.26085 (11)	0.8325 (7)	0.1827 (3)	0.0460 (9)	
C8	0.22240 (19)	0.5394 (9)	0.0718 (5)	0.0307 (12)	
F8	0.26118 (12)	0.5015 (6)	0.0508 (4)	0.0447 (8)	
C9	0.18591 (19)	0.4095 (9)	0.0276 (4)	0.0279 (10)	
H9	0.1861	0.2901	−0.0205	0.033*	

### Atomic displacement parameters (Å^2^)

**Table:**

	*U*^11^	*U*^22^	*U*^33^	*U*^12^	*U*^13^	*U*^23^
Sn1	0.0230 (2)	0.00982 (18)	0.01224 (19)	0.000	0.00759 (19)	0.000
I1	0.02280 (16)	0.01818 (15)	0.01977 (16)	−0.00088 (9)	0.00728 (14)	−0.00114 (10)
I2	0.0304 (2)	0.02089 (19)	0.01304 (19)	−0.00124 (18)	0.00977 (19)	−0.00007 (13)
I3	0.0319 (2)	0.00964 (17)	0.0243 (2)	0.000	0.0134 (2)	0.000
C1	0.029 (3)	0.022 (2)	0.019 (2)	0.0032 (17)	0.0095 (19)	−0.0028 (15)
N2	0.027 (2)	0.022 (2)	0.0249 (19)	−0.0011 (17)	0.0085 (17)	−0.0036 (16)
C3	0.030 (3)	0.029 (2)	0.024 (2)	−0.002 (2)	0.012 (2)	−0.0046 (19)
N4	0.030 (2)	0.023 (2)	0.025 (2)	0.0035 (17)	0.0123 (18)	−0.0023 (16)
C5	0.027 (2)	0.026 (2)	0.0158 (18)	0.0022 (18)	0.0064 (17)	−0.0023 (17)
C6	0.035 (3)	0.026 (2)	0.025 (2)	−0.003 (2)	0.010 (2)	−0.0064 (18)
C7	0.027 (2)	0.034 (3)	0.030 (2)	−0.006 (2)	0.004 (2)	−0.003 (2)
F7	0.0326 (18)	0.055 (2)	0.048 (2)	−0.0165 (17)	0.0106 (16)	−0.0163 (19)
C8	0.024 (3)	0.042 (3)	0.027 (3)	0.005 (2)	0.011 (2)	−0.002 (2)
F8	0.0299 (18)	0.057 (2)	0.050 (2)	0.0018 (16)	0.0168 (19)	−0.0133 (19)
C9	0.034 (3)	0.026 (2)	0.025 (2)	0.001 (2)	0.011 (2)	−0.0035 (18)

### Geometric parameters (Å, º)

**Table:**

Sn1—I1	3.1596 (3)	C3—H3	0.9500
Sn1—I2	3.1129 (1)	N4—C5	1.387 (6)
Sn1—I3	3.1310 (5)	N4—H4	0.75 (5)
Sn1—I3^i^	3.0491 (5)	C5—C6	1.385 (7)
Sn1—I1^ii^	3.1596 (3)	C6—C7	1.361 (7)
C1—C9	1.376 (7)	C6—H6	0.9500
C1—N2	1.378 (7)	C7—F7	1.348 (6)
C1—C5	1.408 (6)	C7—C8	1.404 (7)
N2—C3	1.330 (7)	C8—F8	1.347 (6)
N2—H2	0.95 (6)	C8—C9	1.357 (8)
C3—N4	1.316 (7)	C9—H9	0.9500
			
I1—Sn1—I2	89.374 (6)	C1—N2—H2	131 (3)
I1—Sn1—I2^ii^	90.984 (6)	N4—C3—N2	109.6 (5)
I1—Sn1—I1^ii^	176.539 (14)	N4—C3—H3	125.2
I2—Sn1—I2^ii^	168.154 (12)	N2—C3—H3	125.2
I1—Sn1—I3	91.731 (7)	C3—N4—C5	109.7 (4)
I2—Sn1—I3	84.077 (6)	C3—N4—H4	125 (5)
I1—Sn1—I3^i^	88.269 (7)	C5—N4—H4	124 (5)
I2—Sn1—I3^i^	95.923 (6)	C6—C5—N4	132.3 (4)
I3—Sn1—I3^i^	180.0	C6—C5—C1	122.3 (5)
I3^i^—Sn1—I2^ii^	95.923 (6)	N4—C5—C1	105.3 (4)
I2^ii^—Sn1—I3	84.077 (6)	C7—C6—C5	114.9 (4)
I3^i^—Sn1—I1^ii^	88.270 (7)	C7—C6—H6	122.6
I2—Sn1—I1^ii^	90.984 (6)	C5—C6—H6	122.6
I2^ii^—Sn1—I1^ii^	89.374 (6)	F7—C7—C6	119.9 (5)
I3—Sn1—I1^ii^	91.730 (7)	F7—C7—C8	117.4 (5)
Sn1^iii^—I2—Sn1	180.0	C6—C7—C8	122.7 (5)
Sn1^iv^—I3—Sn1	180.0	F8—C8—C9	121.0 (5)
C9—C1—N2	131.9 (4)	F8—C8—C7	116.3 (5)
C9—C1—C5	121.7 (5)	C9—C8—C7	122.7 (5)
N2—C1—C5	106.3 (4)	C8—C9—C1	115.6 (5)
C3—N2—C1	109.1 (4)	C8—C9—H9	122.2
C3—N2—H2	119 (3)	C1—C9—H9	122.2
			
C9—C1—N2—C3	−179.2 (6)	C1—C5—C6—C7	−0.8 (7)
C5—C1—N2—C3	0.5 (6)	C5—C6—C7—F7	−178.7 (5)
C1—N2—C3—N4	−0.5 (6)	C5—C6—C7—C8	0.3 (8)
N2—C3—N4—C5	0.2 (6)	F7—C7—C8—F8	0.4 (8)
C3—N4—C5—C6	178.4 (5)	C6—C7—C8—F8	−178.7 (5)
C3—N4—C5—C1	0.1 (6)	F7—C7—C8—C9	179.2 (5)
C9—C1—C5—C6	0.9 (8)	C6—C7—C8—C9	0.2 (9)
N2—C1—C5—C6	−178.9 (4)	F8—C8—C9—C1	178.7 (5)
C9—C1—C5—N4	179.4 (5)	C7—C8—C9—C1	−0.2 (8)
N2—C1—C5—N4	−0.4 (5)	N2—C1—C9—C8	179.4 (5)
N4—C5—C6—C7	−178.9 (5)	C5—C1—C9—C8	−0.4 (8)

Symmetry codes: (i) *x*, *y*+1, *z*; (ii) −*x*, *y*, −*z*+1/2; (iii) −*x*, −*y*, −*z*; (iv) *x*, *y*−1, *z*.

### Hydrogen-bond geometry (Å, º)

**Table:**

*D*—H···*A*	*D*—H	H···*A*	*D*···*A*	*D*—H···*A*
N2—H2···I1^v^	0.95 (6)	2.79 (6)	3.610 (4)	145 (4)
N4—H4···I1^i^	0.75 (5)	2.88 (6)	3.587 (4)	157 (6)

Symmetry codes: (i) *x*, *y*+1, *z*; (v) *x*, −*y*, *z*−1/2.
